# Early Warning for Continuous Rigid Frame Bridges Based on Nonlinear Modeling for Temperature-Induced Deflection

**DOI:** 10.3390/s24113587

**Published:** 2024-06-02

**Authors:** Liangwei Jiang, Hongyin Yang, Weijun Liu, Zhongtao Ye, Junwen Pei, Zhangjun Liu, Jianfeng Fan

**Affiliations:** 1 School of Civil Engineering and Architecture, Wuhan Institute of Technology, Wuhan 430073, China; 2 State Key Laboratory of Bridge Intelligent and Green Construction, Wuhan 430034, China; 3 China Construction Third Engineering Bureau Group Co., Ltd., Wuhan 430070, China; 4 Wuhan Mafangshan Engineering Structure Testing Co., Ltd., Wuhan 430070, China

**Keywords:** structural health monitoring, early warning, continuous rigid frame bridges, temperature-induced response, bridge deflection, nonlinear modeling, temperature gradient

## Abstract

Bridge early warning based on structural health monitoring (SHM) system is of significant importance for ensuring bridge safe operation. The temperature-induced deflection (TID) is a sensitive indicator for performance degradation of continuous rigid frame bridges, but the time-lag effect makes it challenging to predict the TID accurately. A bridge early warning method based on nonlinear modeling for the TID is proposed in this article. Firstly, the SHM data of temperature and deflection of a continuous rigid frame bridge are analyzed to examine the temperature gradient variation patterns. Kernel principal component analysis (KPCA) is used to extract principal temperature components. Then, the TID is extracted through wavelet transform, and a nonlinear modeling method for the TID considering the temperature gradient is proposed using the support vector machine (SVM). Finally, the prediction errors of the KPCA-SVM algorithm are analyzed, and the early warning thresholds are determined based on the statistical patterns of the errors. The results show that the KPCA-SVM algorithm achieves high-precision nonlinear modeling for the TID while significantly reducing the computational load. The prediction results have coefficients of determination above 0.98 and fluctuate within a small range with clear statistical patterns. Setting the early warning thresholds based on the statistical patterns of errors enables dynamic and multi-level warnings for bridge structures.

## 1. Introduction

Continuous rigid frame bridges possess significant longitudinal flexural rigidity and lateral-torsional rigidity, making them well-suited for accommodating large spans. This bridge type is a robust structural form that meets considerable force demands. Bridges inevitably experience performance degradation throughout their operation [1,2]. Therefore, the implementation of early warning systems is considered crucial for ensuring the safe operation of bridges [3,4]. Many bridges have been equipped with SHM systems in recent years, providing essential raw data for early warning [5,6,7]. However, extracting indicators that reflect changes in structural performance from vast amounts of SHM data remains a challenging and promising research topic.

Common monitoring indicators in bridges include deflection [8], stress [9,10], bearing displacement [11], etc. Structural responses induced by temperature exhibit low-frequency characteristics and can be used to evaluate bridge performance degradation [12,13]. Numerous scholars have used time and frequency domain analyses to extract temperature-induced deflection (TID) from deflection monitoring signals. A TVFEMD-PE-KLD method based on multi-scale effects was proposed by Li et al. [14], improving the TID separation accuracy. Zhang et al. [15] employed local outlier correction and an S-G convolution smoothing method to extract the TID in the time domain. The foundation for further establishing correlation models between temperature and the TID has been laid by these studies.

Analytical solutions have been explored to reveal the mechanism of the TID [16,17,18], but variations in the TID exist across different bridge types, and the application of analytical solutions is limited by the complexity of actual bridge projects [12]. Measured signals from the SHM systems have been used to analyze the relationship between temperature and the TID, which has become a popular research trend in recent years. Multiple linear regression (MLR) was used to model the deflection of steel truss arch bridges, enabling the detection and location of bearing damages [19]. However, it is a challenge to model the structural temperature-induced responses of concrete box girder bridges using the linear regression method due to the pronounced time-lag effect [20]. Neural network algorithms excel at establishing nonlinear mapping relationships between independent and dependent variables. Yue et al. [21] developed a nonlinear mapping model between temperature and the TID using LSTM, which eliminated peak and phase errors. Yue et al. [22] modeled the TID using a Stack-LSTM-CNN model, achieving results with higher reliability and interpretability. However, the application of these algorithms is limited by the huge computational burden.

Bridge deflection is caused to fluctuate within a certain range by temperature loads, and under extreme conditions, the amplitude may exceed that caused by live loads [8]. Temperature across the same bridge section is correlated; however, temperature gradients within the bridge section will be led by the uneven sunlight and material properties. The static and dynamic characteristics of bridge structures will be affected by the temperature gradients [23]. Analyzing and extracting temperature gradients is crucial for accurately modeling the TID. The Principal Component Analysis (PCA) algorithm was used by Xia et al. [24] to extract major temperature characteristics that reflect spatial temperature information, establishing a regression relationship between temperature and the TID of a cable-stayed bridge. However, limited studies have focused on analyzing and extracting temperature gradients of concrete box girder bridges using the SHM data.

Early warning for bridges is seen as an effective means of ensuring safe operation [25,26]. By leveraging the vast monitoring data provided by the SHM systems, structural information can be extracted to accurately predict future responses of bridges and achieve early warnings [27,28]. The expected health state of the bridge is reflected by the TID prediction model established based on monitoring data corresponding to past time series, where the prediction error of an accurate model only fluctuates within a small range and exhibits clear statistical patterns [29,30]. Setting early warning thresholds based on the statistical patterns of the TID prediction results is considered a feasible approach.

Based on the SHM data from the Yellow River Bridge, which is a continuous rigid frame bridge, a safety warning method based on nonlinear modeling of the TID is proposed in this article. This article is organized as follows:

In Section 2, the theory of nonlinear modeling for the TID based on the KPCA-SVM algorithm is introduced, and the rationality of using the TID as an early warning indicator is demonstrated through simulation analysis.

In Section 3, the deflection and temperature data obtained from the SHM system of the Yellow River Bridge are analyzed, and linear regression analysis of the TID is conducted, as well as quantitative explanations of cross-sectional temperature correlation and temperature gradient.

In Section 4, nonlinear modeling and prediction of the TID are conducted based on the KPCA-SVM algorithm, and a comparison is made with the MLR models.

In Section 5, early warning thresholds for the bridge are determined based on the statistical patterns of the prediction results, and a multi-level warning method is introduced.

## 2. Theory

### 2.1. Nonlinear Modeling for the TID Based on the KPCA-SVM

#### 2.1.1. Kernel Principal Component Analysis

Linearly inseparable samples in a low-dimensional space can be mapped to a high-dimensional space by the KPCA. The PCA is performed in the high-dimensional space, and then, the results back to the original low-dimensional space are projected, thereby obtaining a reduced dimensionality representation of nonlinear samples [31].

Multidimensional nonlinear temperature monitoring data from different measurement points on the same cross-section can be handled by the KPCA, reducing the correlation of temperature monitoring values at various points. New possibilities for developing models that predict structural temperature effects based on temperature monitoring values in the field of civil engineering are offered by the KPCA.

Assuming the dimensionality of the temperature monitoring values is *n* and the number of sampling points in each dimension is *N*, the cross-sectional temperature monitoring data **X** can be represented as $xi\in R^{n}(i=1,2,\ldots ,N)$.

The key of the KPCA lies in mapping linearly inseparable samples *x_i_* in a low-dimensional space to a high-dimensional space, making them linearly separable. The covariance matrix **C** of the high-dimensional space samples can be expressed as follows:

(1) $$C=\frac{1}{N-1}\sum_{i=1}^{N}\phi (x_{i})\phi {\left(x_{i}\right)}^{T}$$

where ϕ is a nonlinear mapping function, which is implicit. 

Therefore, a kernel function K, which can be calculated based on sample data, is introduced for computation:

(2) $$K(x_{i},x_{i})=\langle \phi (xi),\phi {\left(xi\right)}^{T}\rangle$$

where 〈·〉 represents the inner product operation. The result of the calculation is named the kernel matrix **M**.

The greater the variance, the more information the data contains. The core concept of principal component analysis is to find the characteristic distribution of the data with the largest variance. To find the vector *v* with the largest variance, the above problem can be transformed into an eigenvalue solution, and the eigenvalues of matrix **C** and kernel matrix **M** are identical, namely,

(3) Mv=λv

Calculate the eigenvalues and eigenvectors, and sort the eigenvalues in descending order. The maximum eigenvalue corresponds to the first principal component. Select the number of principal components *s* (*s* < *n*) for dimensionality reduction using the cumulative percent variance V*_cp_*:

(4) $$V_{cp}=\frac{\sum_{n=1}^{s}\lambda_{n}}{\sum_{n=1}^{n}\lambda_{n}}\times 100\%$$

The kernel principal components after dimensionality reduction can be expressed as

(5) $$X_{s}^{\prime }=Xv_{s}$$

#### 2.1.2. Support Vector Machine

The SVM is a classic machine-learning algorithm used for classification and regression. Finding an optimal hyperplane in the feature space that distinguishes between positive and negative samples is the core concept of the SVM, and the hyperplane also ensures that the distance from the data points nearest to the hyperplane is maximized. 

The optimal hyperplane is achieved by optimizing the following objective function (OBJ):

(6) $$\{\begin{array}{l} \underset{w,b,\varepsilon }{min}\frac{1}{2}{\left\|w\right\|}^{2}+H\sum_{i=1}^{q}\varepsilon_{p}\\ s.t.\;y_{p}-(w\cdot x_{p}+b)\leq \varepsilon_{p};\;(w\cdot x_{p}+b)-y_{p}\leq \varepsilon_{p} \end{array}$$

where *x_p_* is the input feature vector, and *y_p_* is the corresponding target value; *w* and *b* are the normal vector and intercept of the hyperplane, respectively; ε is the slack variable to handle errors and distribution of data points; and *H* is the regularization parameter to control the complexity of the regressor.

The principal components of the temperature are used as inputs, and the TID is used as the output for the SVM. By solving the OBJ, the optimal hyperplane parameters of the SVM can be obtained, enabling regression analysis of the TID. Compared to traditional regression methods, the SVM offers greater robustness and generalization ability in dealing with complex data distributions [32].

### 2.2. Sensitivity Analysis of the TID to Bridge Degradation

The TID serves as a sensitive indicator of performance degradations of continuous rigid frame bridges. As noted in references [33,34], the long-term deflection of continuous rigid frame bridges is significantly affected by the uneven shrinkage effect of bridge sections caused by temperature gradients, and the structural performance is also impacted by temperature variations.

**Figure 1:**
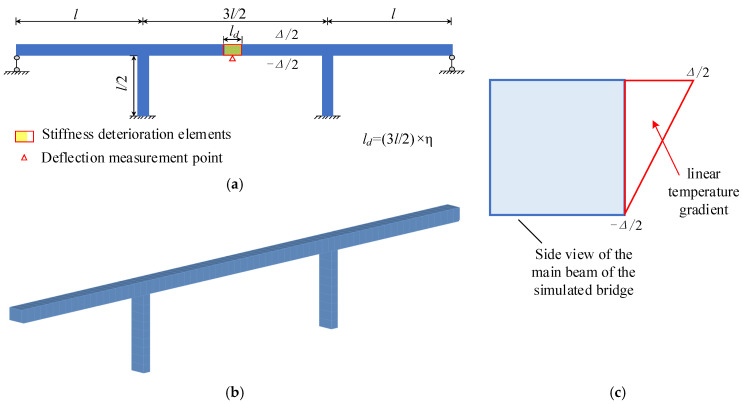
The simulated bridge: (**a**) The elevation of the bridge; (**b**) the finite element model of the bridge; (**c**) the temperature gradient load.

The vertical displacement of bridges increases as the flexural stiffness (*EI*) of bridges decreases [35,36]. The sensitivity of the TID in vertical to *EI* degradation is quantitatively described through a finite element simulation of a 3-span continuous rigid frame bridge. As shown in Figure 1, there are *EI* degradation elements at the mid-span of the bridge, and the ratio of the length of the degradation area to the length of the mid-span is defined as η.

The coefficient of variation D_er_ is used to describe the sensitivity of the TID vertically to the *EI* degradation of bridge performance, and it can be expressed as follows:

(7) $$D_{\mathrm{er}}(l)=\frac{{\mathrm{TID}}_{l,\mathrm{damage}}-{\mathrm{TID}}_{l,\mathrm{normal}}}{{\mathrm{TID}}_{l,\mathrm{normal}}}\times 100\%$$

where the TID*_l_*_,damage_ and TID*_l_*_,normal_ are the TID before and after the degradation of the bridge, respectively.

**Figure 2:**
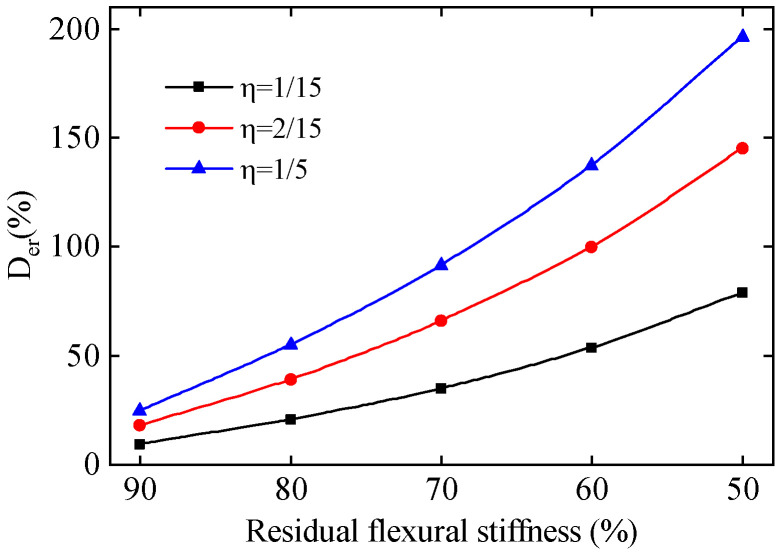
The coefficient of variation of the TID in different residual flexural stiffness.

A temperature gradient load Δ with a value of 5 °C varying linearly along the height is applied to the main beam of the continuous rigid frame bridge, and the distribution of the temperature gradient load is shown in Figure 1c. The D_er_ of the TID in different residual *EI* cases is shown in Figure 2.

It can be seen from Figure 2 that the D_er_ significantly increases as the η increases. The slope of the D_er_ gradually increases with the decrease in the residual *EI*. The results of the simulation analysis highlight the high sensitivity of the TID to both the length range and severity of stiffness degradation in continuous rigid frame bridges.

Note that the specific value of the D_er_ depends on the arrangement and material properties of continuous rigid frame bridges. The simulation analysis above reflects the pattern that significant changes in the TID will be caused by the bridge stiffness degradation. Therefore, the TID can be used as an early warning indicator for the performance of continuous rigid frame bridges. The specific form of bridge damage should be combined with on-site manual inspection and other ways to determine and then take repair and reinforcement measures to ensure the safe operation of the bridge.

### 2.3. Flow Chart for the Proposed Early Warning Method

To achieve early warnings for bridges and ensure safe operation, a bridge early warning method based on the nonlinear modeling for the TID is proposed in this article, which can be divided into the following five steps:

(1) Preprocess the data from the SHM system to obtain temperature monitoring values and deflection monitoring values, and use wavelet decomposition techniques to extract the TID.
(2) Perform kernel principal component analysis on the temperature monitoring values to extract the principal components that reflect temperature gradient information across the bridge cross-section.
(3) Use the results of kernel principal component analysis as inputs and the TID as outputs of the SVM to conduct a regression analysis of deflection responses under temperature influence.
(4) Set warning thresholds based on the statistical characteristics of the TID prediction errors to achieve early warnings for bridges.
(5) When the early warning is triggered, take repair and reinforcement measures to bring the bridge back to an intact state, and replace the measured TID with the predicted TID during the process of repair and reinforcement.

**Figure 3:**
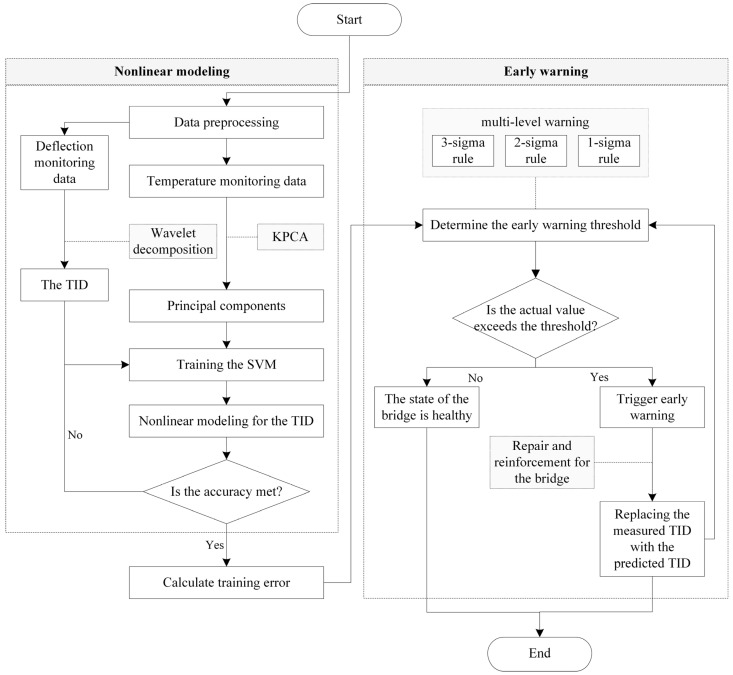
Flowchart for the proposed early warning method.

The flowchart for the proposed method is shown in Figure 3.

## 3. Introduction of the SHM System and Data Analysis

### 3.1. Health Monitoring System of the Yellow River Bridge

**Figure 4:**
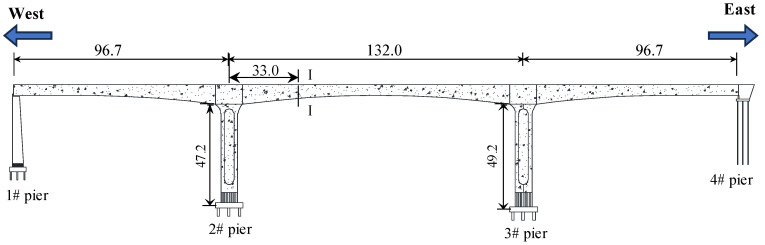
The elevation of the Yellow River Bridge (unit: m).

The Yellow River Bridge is located within the territory of Qingshuihe County, Inner Mongolia Autonomous Region, China. As shown in Figure 4, it has a latitude of approximately 39.8° north and runs in an east–west direction. A variable-section prestressed concrete continuous beam with spans of (96.7 + 132.0 + 96.7) meters is used for the main beam, featuring a single-box, single-chamber structure. Rectangular hollow piers with a variable-width design in the transverse direction are adopted for the rigid abutment piers, among which 2# Pier has a height of 47.2 m and 3# Pier has a height of 49.2 m.

**Figure 5:**
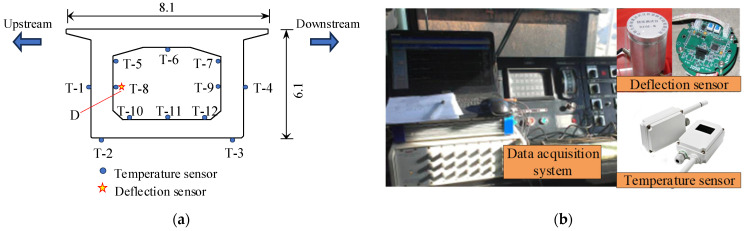
Layout of sensors and the data acquisition system: (**a**) layout of sensors (unit: m); (**b**) data acquisition system.

As shown in Figure 5, an SHM system was equipped during the initial operation of the Yellow River Bridge. Twelve temperature sensors (T-1 to T-12) were arranged on the top, bottom, and sides of the I-I cross-section, and one deflection sensor (D) was installed on the inner side of the web. The sampling frequencies of the deflection sensor and temperature sensors are 1 Hz and 1/600 Hz, respectively. The bridge deflection is monitored using a digital deflection sensor of type BDM-II, which has a testing accuracy of 0.1 mm.

### 3.2. Analysis for Deflection Data

**Figure 6:**
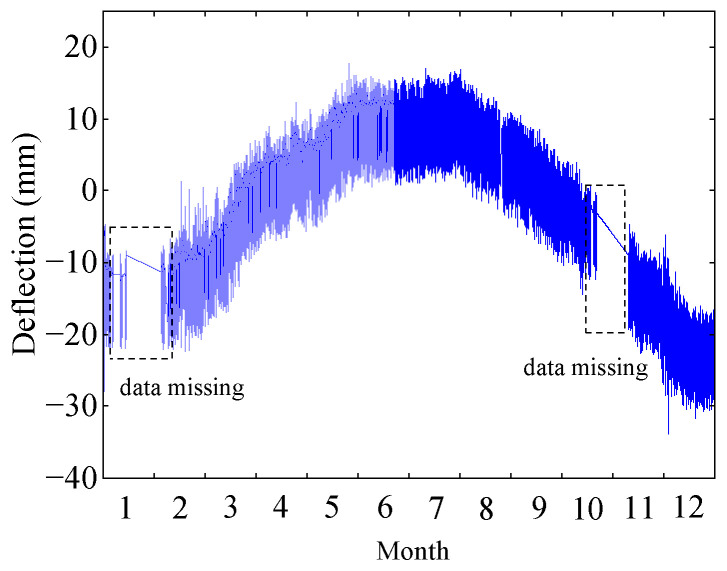
Deflection monitoring data.

The deflection monitoring data of the I-I cross-section of the Yellow River Bridge in 2014 are shown in Figure 6. Due to equipment malfunctions in the SHM system, deflection monitoring values are partially missing, which have been addressed by performing linear interpolation to complete them. It is clear that the TID dominates the deflection monitoring data over a one-year time span.

**Figure 7:**
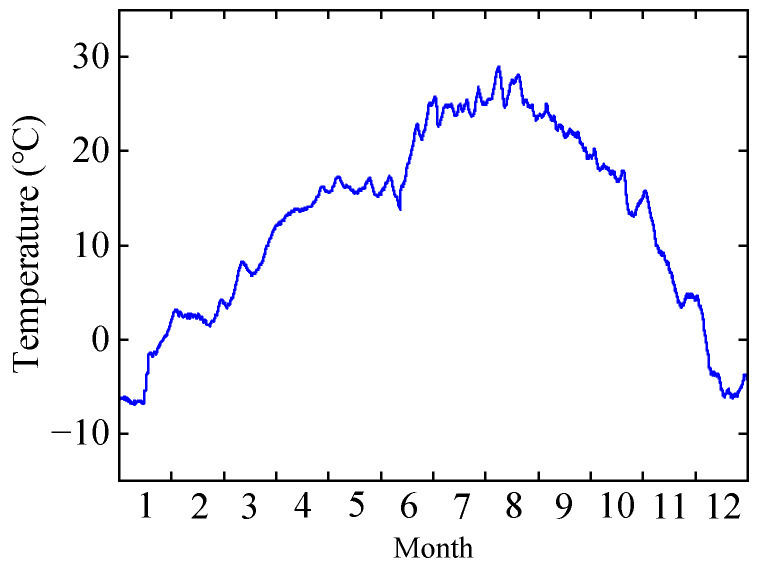
Structural temperature.

To analyze the correlation between bridge deflection and temperature, the structural temperature is shown in Figure 7 (measured at T-8). It can be observed that the interpolated deflection monitoring values align with the trend in structural temperature changes, indicating a correlation between the bridge deflection and the temperature.

**Figure 8:**
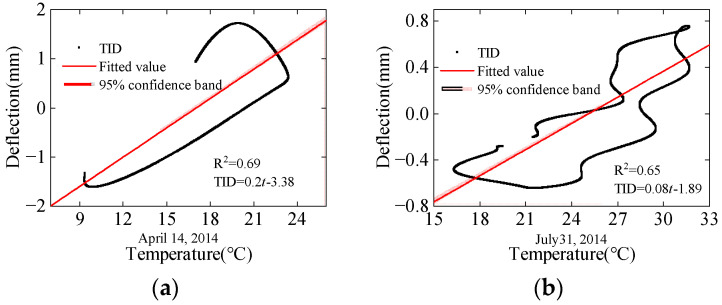

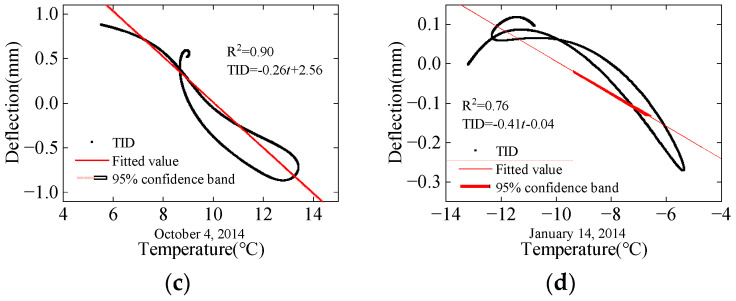
Linear regression analysis of the TID: (**a**) spring; (**b**) summer; (**c**) autumn; (**d**) winter.

The results of linear regression analysis between the TID and structural temperature of different seasons are shown in Figure 8. Due to the time-lag effect, the regression analysis results form a circular pattern. The coefficient of determination is used to assess the accuracy of the regression models, which can be expressed as follows:

(8) $$R^{2}=1-\frac{\sum_{a=1}^{g}{\left(y_{a}^{\mathrm{TID}}-y_{a}^{L}\right)}^{2}}{\sum_{a=1}^{g}{\left(y_{a}^{L}-\bar{y}\right)}^{2}}$$

where *g* is the number of sample points; $y_{a}^{\mathrm{TID}}$ is the actual value of the TID; $y_{a}^{L}$ is the value calculated by the linear regression models; and $\bar{y}$ is the average value of the TID.

The accuracy of the linear regression models varies significantly across different seasons. In winter and spring, where temperature fluctuations are relatively small, the linear regression performs well, with coefficients of determination of 0.76 and 0.69, respectively. However, in summer, due to significant temperature fluctuations, the time-lag effect leads to a poorer result in linear regression analysis, with a coefficient of determination of only 0.65. In autumn, with the smallest daily temperature difference, the coefficient of determination is the highest among the four seasons, reaching 0.90.

The complex temperature distribution and time-lag effect of bridges make it very difficult to describe the variation pattern of the TID through linear regression. Choosing regression models with stronger generalization abilities for modeling and selecting physical quantities that better represent the temperature distribution pattern of bridge sections to describe the nonlinear relationship between temperature and the TID is necessary.

### 3.3. Cross-Section Temperature Gradient Analysis

#### 3.3.1. Patterns of Temperature Variation

**Figure 9:**
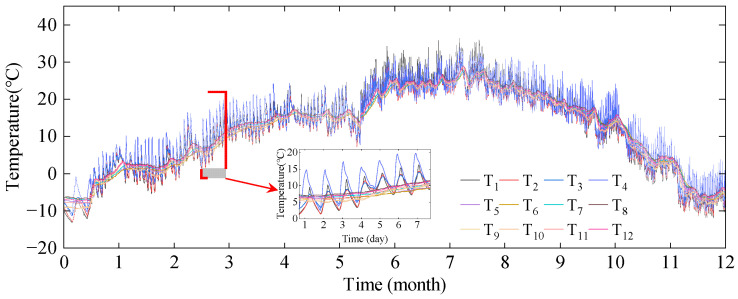
Temperature monitoring data of the I-I section of the Yellow River Bridge.

The temperature monitoring data of the I-I section of the Yellow River Bridge over one year is shown in Figure 9. The cross-sectional temperature fluctuates in sinusoidal function patterns with different periods over one year and one day, respectively. Temperature monitoring values at different positions on the same section exhibit two types of fluctuation patterns: outside and inside of the box. Overall, the temperature outside the concrete box girder bridge exhibits larger absolute values and more drastic fluctuations, while the temperature inside the box shows smaller absolute values and smoother fluctuations. The temperature variations inside and outside of the box follow similar overall trends, but the peak temperatures at different measurement points occur at significantly different times, indicating a pronounced cross-section temperature gradient.

**Figure 10:**
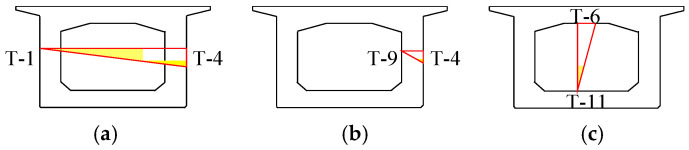
Schematic diagrams of temperature gradients: (**a**) tg_1_; (**b**) tg_2_; (**c**) tg_3_.

**Figure 11:**
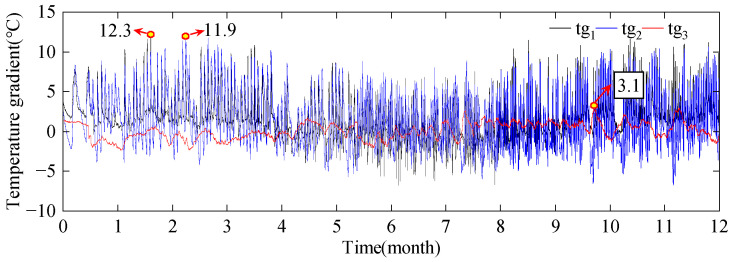
The variation pattern of temperature gradients.

Analysis of temperature gradients for the lateral exterior of the concrete box girder I-I section (tg_1_), the inner and outer sides of the sunlit lateral webs (tg_2_), and the interior of the chamber (tg_3_) is conducted. Schematic diagrams of temperature gradients are shown in Figure 10. The variation patterns of temperature gradients are shown in Figure 11. There are significant differences in temperature gradients at different positions within the same cross-section. The temperature gradients for the lateral exterior and the inner and outer sides of the sunlit lateral webs fluctuate dramatically, with maximum temperature gradients of 12.3 °C and 11.9 °C, respectively. In contrast, the temperature gradients for the top and bottom plates inside the chamber fluctuate gently, with a maximum temperature gradient of only 3.1 °C. And there is no clear pattern of the peak times of temperature gradients at different positions.

#### 3.3.2. Temperature Correlation Analysis

There is a correlation between temperature monitoring values at different measurement points on the same cross-section of the bridge, and the Pearson correlation coefficient *r* is used to quantitatively describe the correlation; *r* can be expressed as follows:

(9) $$r=\frac{1}{h-1}\sum_{j=1}^{h}(\frac{X_{1,j}-\bar{X_{1}}}{\sigma_{X_{1}}})(\frac{X_{2,j}-\bar{X_{2}}}{\sigma_{X_{2}}})$$

where *h* is the number of temperature monitoring data; $\bar{X_{1}}$ and $\bar{X_{2}}$, $\sigma_{X_{1}}$ and $\sigma_{X_{2}}$ are the average value and standard deviation of temperature at different measurement points, respectively.

**Figure 12:**
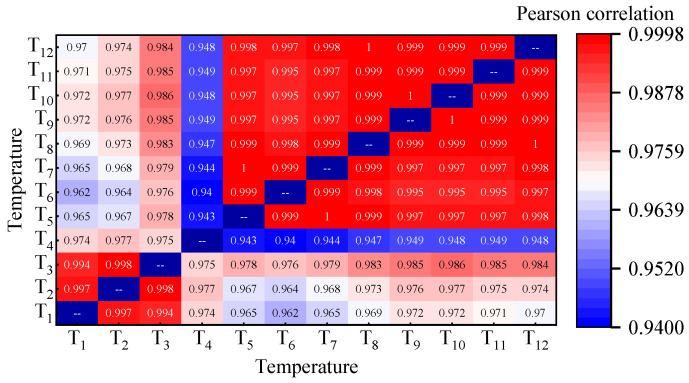
The correlation heatmap of temperature for the I-I cross-section.

The correlation heatmap of the temperature at different points is shown in Figure 12. The *r* of temperature inside of the box is above 0.99, indicating a high level of consistency and synchronization in temperature variations inside the box. For temperature outside of the box, influenced by direct sunlight radiation and other environmental factors such as wind and weather conditions, the *r* is slightly lower. Located on the sunlit side, the T-4 monitoring point exhibits the lowest correlation coefficient but still exceeds 0.94, which indicates that there is an obvious correlation between the temperatures inside and outside of the bridge box.

The temperature monitoring values of the bridge section exhibit both correlation and differences, resulting in significant temperature gradients across the section. Influenced by environmental changes, it is challenging to accurately quantify the distribution pattern of temperature gradients across different seasons. Meanwhile, the temperature monitoring values across the section show a strong correlation, indicating a substantial redundancy of information obtained from the SHM system, which poses a significant computational burden for modeling the TID based on actual temperature measurements. Therefore, it is necessary to extract the variation pattern of temperature gradient through a new method.

## 4. Nonlinear Modeling for the TID

### 4.1. Temperature Principal Component

**Figure 13:**
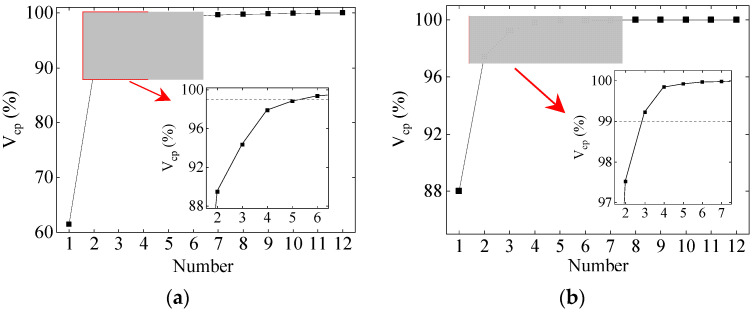
The results of temperature principal components analysis: (**a**) July; (**b**) December.

The temperature monitoring values at different points on the bridge section can reflect the variation pattern of section temperature. However, due to the strong correlation of temperature within the time domain for the same cross-section, the information on temperature gradients is masked, resulting in a significant computational burden for modeling the TID based on the SHM data. Principal components obtained by the KPCA can accurately express the information of the original data, reducing redundancy in monitoring data and obtaining information on cross-section temperature gradients. Analysis was conducted for representative periods of the Yellow River Bridge (July and December), and the results of temperature principal components are shown in Figure 13.

As shown in Figure 13, *V_cp_* reaches 99% when the first five and three principal components are selected in July and December, respectively. Compared to the original 12-dimensional data, the data volume is reduced by 58% and 75%, respectively, and the computational burden of nonlinear modeling for the TID is significantly reduced. The reduction in the dimensionality of monitoring data provides the possibility of constructing prediction models between temperature and the TID.

### 4.2. Nonlinear Modeling for the TID

**Figure 14:**
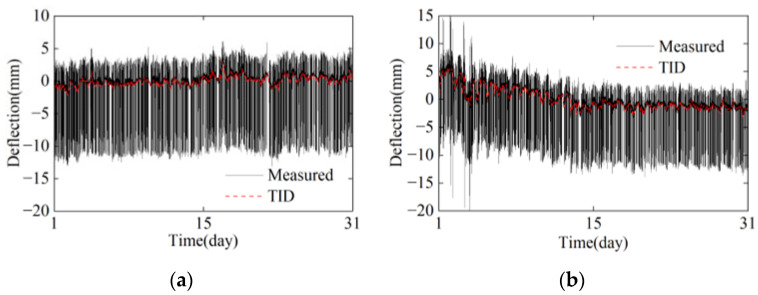
Temperature-induced deflection: (**a**) July; (**b**) December.

In the SHM signals of bridge deflection, the TID exhibits long-wave and low-frequency characteristics, whereas the deflections caused by the train loads have opposite characteristics and exhibit short-wave high-frequency characteristics. The TID can be extracted based on wavelet transform in the frequency domain [37]; the TID of July and December are shown in Figure 14. The TID varies relatively smoothly over a one-month time span, and the deflection monitoring values are dominated by train loads.

**Figure 15:**
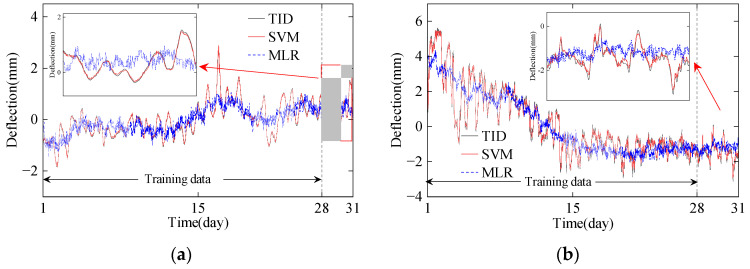
The predicted results of the TID: (**a**) July; (**b**) December.

Using the temperature principal components as inputs and the TID as outputs, prediction models based on the SVM are trained for nonlinear modeling. The dataset is split into training and validation sets in a 9:1 ratio. Considering that the temperature input is multidimensional, the results obtained from the MLR models are used as comparisons. The predicted TID for different periods is shown in Figure 15.

The accuracies of different models are listed in Table 1, where RMSE is the root mean squared error and MAE is the mean absolute error. The RMSE and the MAE can be, respectively, expressed as follows:

(10) $$\mathrm{RMSE}=\sqrt{\frac{1}{g}\sum_{c=1}^{g}{\left(y_{c}^{\mathrm{DIT}}-y_{c}^{R}\right)}^{2}}$$

(11) $$\mathrm{MAE}=\frac{1}{g}\sum_{c=1}^{g}\left|y_{c}^{\mathrm{DIT}}-y_{c}^{R}\right|$$

where $y_{c}^{R}$ is the value of the TID calculated by the regression models.

As shown in Figure 15, it is evident that the KPCA-SVM algorithm achieves higher prediction accuracy. As listed in Table 1, the performance of the MLR varies across different seasons, with better results in winter (December) where overall temperature fluctuations are gentler, yielding a correlation coefficient of 0.78. However, in summer (July), when the overall temperature fluctuations across the bridge section are larger, the MLR prediction results have a lower correlation coefficient of only 0.47. Additionally, the MLR struggles to describe extreme temperature changes, as the peak times of the predicted results differ significantly from the TID, indicating the inability of MLR to overcome the time-lag effect.

**Figure 16:**
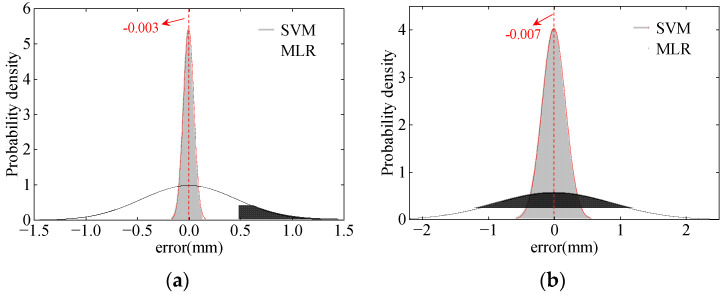
The probability density of the prediction errors: (**a**) July; (**b**) December.

Using principal components of temperature as inputs and considering cross-section temperature gradients, the peak TID can be accurately predicted by the nonlinear KPCA-SVM algorithm. The correlation coefficient of the prediction results exceeds 0.98, and both RMSE and MAE are lower than the MLR. The probability density of prediction errors of the TID is shown in Figure 16.

It can be seen from Figure 16 that using principal components reflecting section temperature gradients as inputs for nonlinear modeling of the TID, 99.9% and 98.8% of the prediction results have absolute errors less than 0.5 mm in July and December, respectively. Therefore, accurate data support can be provided by the KPCA-SVM algorithm for bridge early warning.

## 5. Early Warning for Bridges

In 2014, the Yellow River Bridge was in its early operational stage and intact condition. The prediction models for the TID were established using measured signals as training samples, reflecting the TID variation patterns associated with the intact condition of the bridge.

Further analysis of the probability density chart shows that the mean training errors in July and December are −0.003 mm and −0.007 mm, respectively, both of which are consistent with a 0-mean distribution. This suggests that when the structural performance of the bridge is intact, the prediction error for TID based on the KPCA-SVM algorithm fluctuates within a small range. And when structural performance deteriorates, the prediction error changes significantly. Therefore, it is reasonable to use the statistically regular training errors to determine the early warning thresholds.

### 5.1. Selection of the Warning Threshold

The key to bridge structural early warning lies in the selection of warning thresholds. In this study, the warning thresholds are determined based on the probability density of prediction errors. The upper warning threshold (UWT) and lower warning threshold (LWT) can be defined as follows:

(12) Φ(UWT≥δ≥LWT)=β

(13) $$\alpha =\Phi^{-1}(\beta )$$

(14) $$\mathrm{UWT}=E(x^{\prime })+\alpha \sigma_{e}$$

(15) $$\mathrm{LWT}=E(x^{\prime })-\alpha \sigma_{e}$$

where Φ is the cumulative distribution function (CDF); δ is the margin of safety for the TID; β is the significance level for early warning, which is set by the bridge owner based on the condition of the bridge; $E(x^{\prime })$ is the predicted TID; *α* is the standard deviation amplification factor determined by the inverse function of the CDF; and $\sigma_{e}$ is the standard deviation of the prediction errors of the training set.

**Figure 17:**
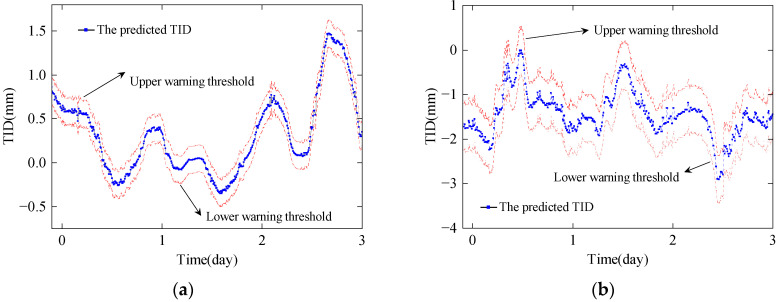
Early warning thresholds of the last four days: (**a**) July; (**b**) December.

Based on the rule of 3-sigma, setting *α* to 3 gives *β* of 99.74%, and the results of the TID early warning thresholds of the last three days of July and December of the Yellow River Bridge are shown in Figure 17.

As shown in Figure 17, the early warning thresholds for the TID dynamically change over time, providing bridge owners with a basis for assessing the condition of the bridge. A multi-level warning system for the bridge condition can be achieved by adjusting α and *β*. Setting *α* to 2 and 1 corresponds to *β* values of 95.44% and 68.26%, respectively. The multi-level warning system allows for more time to respond to potential structural damage to the bridge.

For training errors that do not meet the rule of 3-sigma, the probability density function can be obtained using kernel density estimation methods, and then, *α* and *β* can be determined, through which the applicability of the early warning method proposed in this article is enhanced.

When the early warning system detects bridge damage and sends out warning signals, it should be combined with on-site manual inspections to determine the specific form of damage to the bridge and take repair and reinforcement measures to bring the bridge back to an intact state. For the time between the appearance of damage and the completion of repair and reinforcement, the predicted TID should be used instead of the measured TID for subsequent prediction, which ensures that the samples in the training set always reflect the intact state of the bridge.

### 5.2. Selection of the Training Set

The ratio of the lengths of the training set to the validation set Ω is an important parameter that directly affects the predictive performance and generalization ability of the model. Ω is adjusted to further validate the applicability of the KPCA-SVM algorithm, and the evaluation metrics for the prediction accuracy of the validation set (December) are listed in Table 2.

It can be seen from Table 2 that when Ω is reduced from 9:1 to 5:5 (1:1), the accuracy has not been significantly decreased. When Ω is reduced to 1:1, the R^2^ value remains at a relatively high level of 0.95, and the RMSE indicates that the model does not produce significant outliers. Additionally, the MAE is below 0.1 mm. This demonstrates that the KPCA-SVM-based TID modeling method has a strong generalization ability and can provide data support for bridge early warning. The TID prediction method based on the KPCA-SVM algorithm requires the training and validation sets to be continuous in the time series, and therefore, the prediction of the TID over a longer time can be achieved by expanding the volume of the training set.

## 6. Conclusions

(1) The deflection response of the bridge is consistent with the trend of structural temperature changes, following sinusoidal function patterns over one year and one day. Due to the time-lag effect, the linear regression analysis between temperature and the TID results in a circular pattern. It is difficult for both the simple linear and multiple linear regression to accurately predict the TID.
(2) Temperature monitoring values at different points on the cross-section of the concrete box girder bridge exhibit two forms of variation: inside and outside of the box. The outside box has higher absolute temperatures and greater fluctuations, while the inside box has lower absolute temperatures and smoother fluctuations. The Pearson correlation coefficients of temperature monitoring values from different points are greater than 0.94, but the peak times differ, and there is a significant temperature gradient across the cross-section of the concrete box girder.
(3) Extracting temperature gradient information from the bridge cross-section using the KPCA-SVM algorithm allows for the establishment of an accurate nonlinear TID prediction model while significantly reducing the computational burden. The prediction model achieves the coefficients of determination (R^2^) greater than 0.98. The KPCA-SVM algorithm demonstrates a strong generalization ability, with the correlation coefficient of prediction results remaining above 0.95 even when the ratio of the training set to the validation set is reduced to 1:1.
(4) The TID can be used as an indicator for continuous rigid frame bridge performance degradation. Setting the TID early warning thresholds based on the statistical patterns of KPCA-SVM algorithm training errors enables dynamic warnings for bridges, and the implementation of multi-level warnings can be achieved by a simple adjustment of parameters. A combination of early warning systems and on-site manual inspections provides important safeguards for the safe operation of bridges.

In summary, a new early safety warning method for bridges based on the analysis of temperature and the TID variation patterns for concrete box girder bridges is proposed in this article. A reference for a continuous rigid frame bridge is provided by relevant conclusions. However, due to differences in load-bearing performance across different bridge types, further verifications for the applicability of the conclusions to other bridge types are required.

## Figures and Tables

**Table 1:** The accuracies of different models (validation data).

Model	July			December		
	R^2^	RMSE	MAE	R^2^	RMSE	MAE
SVM	0.99	0.08	0.06	0.98	0.10	0.07
MLR	0.47	0.51	0.40	0.78	0.88	0.68

**Table 2:** Evaluation metrics of the KPCA-SVM with different Ωs.

Ω	R^2^	RMSE	MAE
9:1	0.98	0.093	0.065
8:2	0.98	0.100	0.065
7:3	0.97	0.117	0.074
6:4	0.96	0.126	0.078
5:5	0.95	0.144	0.089

## Data Availability

The data presented in this study are available on the request from the corresponding author.
